# The effect of administration of Del Nido cardioplegia solution containing vitamin C on myocardial protection and clinical outcomes in patients undergoing coronary artery bypass graft surgery

**DOI:** 10.1186/s43044-025-00641-3

**Published:** 2025-07-17

**Authors:** Farshad Jalili Shahandashti, Mohammadhadi Mozayan, Leyla Abdolkarimi, Faranak Kargar, Amene Ghanbari, Saeid Heidarinia, Mohammad Ziae Totonchi Ghorbani, Seyed Salaheddin Nabavi, Seyyed Ebrahim Hosseini zargaz

**Affiliations:** 1 Rajaie Cardiovascular Medical and Research Institute, Shaheed Rajaie Cardiovascular Medical and Research Institute, Tehran, Islamic Republic of Iran; 2 Department of General Surgery, School of Medicine, Imam Khomeini Hospital, Golestan Hospital, Ahvaz Jundishapur University of Medical Sciences, Ahvaz, Islamic Republic of Iran; 3 Cardiovascular Diseases Research Center, Birjand University of Medical Sciences, Birjand, Islamic Republic of Iran

**Keywords:** Cardiopulmonary bypass, CABG, Cardioplegia solution, Vitamin C, Hemodynamic status

## Abstract

**Background:**

A main concern during cardiac surgery is the protection of the heart. Myocardial ischemia tends to increase the generation of reactive oxygen species. Based on its oxidation–reduction potentials, vitamin C is the most powerful antioxidant to counteract the effects of free radicals. This reason made the researcher to perform this study with the goal to determine and evaluate the effect of on perioperative clinical outcomes and laboratory criteria of coronary artery bypass grafting (CABG) cases.

**Methods:**

This randomized clinical trial (RCT) was done in 2022 at Rajaie Cardiovascular Medical and Research Center. Following obtaining permission from the ethics committee, 62 CABG patients were randomly allocated to two groups and were included in the study. The control group received routine Del Nido cardioplegia solution while vitamin C group received Del Nido cardioplegia solution containing 3gr of vitamin C. In this study, demographic information and variants like hemodynamic, blood, and FFP transfusion were evaluated and compared. Data were analyzed by two-way repeated measures ANOVA.

**Results:**

According to the findings, diastolic blood pressure (DBP) in the vitamin C group was lower, which was significant across both groups (*P* < 0.05); however, these alterations were in the normal range. The groups showed no significant difference in other parameters like fresh frozen plasma (FFP) transfusion in the intensive care unit (ICU) and return to baseline heart rate (*P* > 0.05). Also, the average cardiac arrest time (average time to cardiac arrest from the time of cardioplegia injection) in the controls was significantly higher compared to the intervention group (*P* = 0.028).The data indicate that for CK-MB, the group effect is not statistically significant, while the effect of time and the interaction effect of group and time are significant. For troponin, the group effect and the interaction effect are not significant, although the effect of time is significant. Therefore, adding vitamin C to the cardioplegia solution does not significantly affect troponin**.**

**Conclusion:**

One of the factors that contribute to myocardial protection is the reduction in the time to achieve cardiac arrest. In the group that received vitamin C, this time was reduced; therefore, it can be concluded that in this group, the risk of damage due to lack of blood flow and oxygen to the cardiac tissue is lower. *Clinical trial number:* This study is a randomized clinical trial (RCT).IRCTID: IRCT20220716055477N1.

## Background

Coronary artery bypass grafting (CABG) is a commonly used cardiac surgery [1]. In Iran, over 50 000 open-heart surgeries are conducted each year, of which approximately 50–60% are CABG procedures [2]. According to a study, approximately 340,000 CABG surgeries are conducted every year in the USA, making this procedure one of the most common cardiac surgeries. Most of these surgeries are performed on the pump in such a way that a cardioplegic solution is used to arrest the heart, and circulation is temporarily maintained via cardiopulmonary bypass (CPB).

A primary concern during cardiac surgery is heart protection. Optimal cardiac protection during the ischemic period, which indeed is a period when the aorta is clamped, is achieved by inducing the heart into cardiac arrest status. This is accomplished through the use of a solution called cardioplegia. Cardioplegia aims to induce rapid cardiac arrest in diastole, resulting in a quiet and relaxed heart. This facilitates surgery and represents a phase where myocardial metabolism is often at its lowest. However, despite cardiac inactivity with cardioplegic solution administration, cardiac oxygen, and energy consumption never reaches zero.

Therefore, due to ischemia and the assembly of metabolic waste products, the possibility of cellular damage on a smaller scale always exists, and as the protective impact of the cardioplegic solution on the heart is restricted in duration, repeated injections at specific time intervals are necessary. Further reduction in the metabolic state of the heart is achieved by cooling through the injection of a cold cardioplegic solution and central body cooling [3].

Myocardial protection during ischemia and reperfusion is one of the main issues during cardiac surgery and the cardioplegia, defined as the temporary cessation of myocardial contractions, and is an essential part of cardiopulmonary bypass (CPB). Cardioplegia has a significant impact on preventing inflammatory responses, ischemia, and reperfusion [4, 5].

Injuries resulting from ischemia and reperfusion are categorized into two distinct groups: reversible and irreversible. Reversible injuries are manifested by transient decreases in cardiac function, myocardial edema, and functional recovery without leaving long-term complications. Irreversible cardiac injuries involve myocardial apoptosis or necrosis, leading to electrocardiographic changes; the release of cardiac-specific enzymes, such as creatine phosphokinase (CPK) or troponin, into the bloodstream; and residual ventricular dysfunction in hypokinetic or dyskinetic segments [6].

Oxidation is a chemical process that can generate free radicals, which initiate chain reactions that may harm living cells. Antioxidant agents are substances that halt these chain reactions [7]. Oxidative stress (OS) refers to the imbalance between the generation of oxidants, primarily reactive metabolites and free radicals, and the body’s capability in neutralizing them through its defense mechanisms [8]. To manage OS, both plants and animals rely on intricate systems of overlapping antioxidants, including endogenous compounds like glutathione and enzymes (such as catalase and superoxide dismutase), as well as dietary antioxidants like vitamins C and E [7]. Under extreme conditions, such as trauma, surgery, ischemia, reperfusion, and infection, the generation of ROS increases, often surpassing the capacity of the body’s natural antioxidants. This imbalance leads to damage to macromolecules, including lipids, proteins, carbohydrates, and nucleic acids, which disrupts their biological functions and significantly contributes to cellular damage and organ dysfunction [9].

Ischemia creates conditions for the production of oxygen radicals and the resulting damage. As shown by a clinical study by Prasad et al., oxygen radicals are released during and 24 h after the end of CPB in patients undergoing CABG surgery [10]. During surgery, the myocardium is vulnerable to damage induced by ROS at several stages: (1) initiation of cardioplegic injection, (2) cardioplegic reperfusion after multiple doses of cardioplegia, and (3) removing the aortic clamp and initiation of reperfusion following the cardioplegic phase [11].

Vitamin C, as an essential micronutrient, is associated with many biological and biochemical functions. It can be found in plasma in two types: ascorbic acid and its oxidized counterpart, dehydroascorbic acid (DHA), with DHA accounting for less than 10% of the ascorbic acid in human plasma. Humans cannot produce vitamin C due to the absence of the final enzyme required for its biosynthesis. The vitamin is absorbed through the gastrointestinal tract and remains unbound in the plasma, making it dialyzable. As an electron donor, vitamin C serves multiple roles in the body and is crucial for the activity of over sixty enzymes. Additionally, it is associated with the metabolism of bile acids, cholesterol, and steroids [8]. Due to its strong antioxidant properties and redox potential, vitamin C is regarded as the most effective antioxidant for counteracting free radical damage [12]. It helps protect vital organs, including those of the cardiovascular, nervous, and renal systems. Moreover, it affects inflammation, bleeding and coagulation and can prevent organ damage. The accumulating evidence supporting the positive impacts of vitamin C on cardiac surgery has resulted in its widespread use, driven by four factors: (1) its general safety, allowing for broad application; (2) the ability to administer it without dose adjustments; (3) its familiarity to both healthcare providers and patients; and (4) its low cost of production and administration [8, 13, 14].

Given the growing number of these patients in our country and considering the aforementioned materials, limited research has assessed the impacts of adding vitamin C to the cardioplegic solution. The high prevalence of patients undergoing CABG and the significant morbidity and mortality related to these procedures, coupled with the desire to reduce the duration of intensive care unit (ICU) stay and associated healthcare costs, motivated this study. Due to the importance of myocardial protection during ischemia induced by cardioplegic administration, the high prevalence of patients undergoing CABG, the significance of complications, mortality, and duration of ICU stay, and economic costs associated with CABG surgery for patients and the country’s medical system, this research aims to determine the effectiveness of administering of Del Nido cardioplegic solution containing vitamin C on clinical outcomes and laboratory parameters in CABG patients.

## Method

This randomized controlled trial was done on patients who were candidates for CABG with CPB referring to the Cardiovascular Medical, Research, and Educational Center in 2022. In order to conduct this clinical trial, after the approval of the project by the Deputy of Research Cardiovascular Medical, Research, and Educational Center and obtaining the ethics code and research permit from this center, the researcher visited the cardiac surgery ward according to the predetermined schedule.

In order to collect data, all eligible patients signed written informed consent prior to the entrance to the operating room. In the order of the operating room admission, patients were then randomly allocated to the intervention or control group (*n* = 36 per group) using a block randomization method (12 blocks of 6) generated by the CREATE A RANDOMISATION LIST software; 5 patients were excluded from each group based on the exclusion criteria, totaling 31 patients for each group. A total of 62 patients were finally assessed.

All anesthesia and CPB procedures were performed identically for all patients. In patients of each group, after anesthesia induction and median sternotomy, heparin was delivered intravenously at 300 U/kg to prevent clot formation during CPB. Additional heparin was administered intraoperatively to maintain an activated clotting time (ACT) above 400 s, if necessary. Following the complete connection of the patient to the CPB circuit, antegrade cardioplegic solution, Del Nido, which is electronically similar to extracellular fluid and maintained at a temperature of 2–8 °C, was infused into the aortic root after the aortic clamp ) A single dose of del Nido cardioplegia contains 26 mEq/L of potassium chloride, 13 mL of 1% lidocaine, 3.2 g/L of 20% mannitol, 2 g of 50% magnesium sulfate, 13 mEq/L of sodium bicarbonate, and the system pressure of 100–200 mmHg.) [15, 16]. In the intervention group, 3 g of vitamin C was added to the cardioplegic solution. The control group received only the routine Del Nido cardioplegic solution. During surgery, mild hypothermia (32–34 °C) was induced, and mean arterial pressure (MAP) was preserved at 50–80 mmHg [17].

Primary outcomes include pump time, aortic clamp time, cardiac arrest time, volume of blood product transfusion, and hemodynamic parameters, while secondary outcomes include ventilation time, duration of stay in the intensive care unit, duration of stay in the ward, and cardiac enzymes.

Hemodynamic parameters (HR, DBP, SBP, CVP) were measured by monitoring system prior to anesthesia induction, after cardioplegia administration, during rewarming, after the end of CPB, upon ICU admission, and 12 and 24 h postoperatively. Also other parameters included blood transfusion (packed red blood cells), Platelet transfusion and FFP transfusion were measured.

### *The inclusion criteria

- Age between 30 and 70 years
- Left ventricular ejection fraction greater than 30%.
- Cardiac enzymes within normal limits.
- No history of sternotomy and previous cardiac surgeries.
- No history of liver, kidney, or lung diseases.
- No valve problems that require simultaneous surgery with coronary artery procedures.
- No pacemaker, pregnant. History of allergy to vitamin C and no G6PD deficiency.

### *Exclusion criteria

- Pump time exceeding 120 min.
- Cross-clamp time exceeding 100 min.
- Use of ultrafiltration technique during cardiopulmonary bypass.
- Return of the patient to the cardiopulmonary machine for any reason during the surgery.
- In the end, after collecting data, changes in each of these parameters were compared and analyzed in both groups using SPSS version 26.

### Data analysis

Data were analyzed by SPSS 26. Data were introduced as “mean ± standard deviation” or “median (interquartile range [IQR])” for quantitative data and as “frequency (percentage)” for qualitative data.

### Sample size calculation and formula

Based on the study by Emadi and colleagues in 2019 in Shiraz [18], the required sample size was determined using the following formula:

$$n_{1} = \frac{{\left( {Z_{{1 - \frac{\alpha }{2}}} + Z_{1 - \beta } } \right)^{2} \times \left( {\sigma_{1}^{2} + \frac{{\sigma_{2}^{2} }}{k}} \right)}}{{\Delta^{2} }} \ldots n_{2} = k \times n_{1}$$

Considering a 20% dropout rate of patients after applying the exclusion criteria from the study, the required sample size was 36 individuals in each group, totaling 72 CABG candidates needed for this study; 5 patients were excluded from each group based on the exclusion criteria, totaling 31 patients for each group.

To compare the mean scores of quantitative variables (such as age, intubation duration, ICU stay duration, hospital stay duration, etc.) between the vitamin C and control groups, the independent samples t-test and the nonparametric Mann–Whitney U test were used. The normality of the quantitative variables’ frequency distribution was evaluated using the Kolmogorov–Smirnov test and by calculating skewness and kurtosis. Additionally, the Fisher’s exact or Chi-square test compared the frequency of qualitative variables (e.g., gender) between the two groups.

A two-way repeated measures analysis of variance (ANOVA) compared the average scores of LVEF and myocardial injury biomarkers between the vitamin C and control groups across various time points (preoperatively, postoperatively, upon ICU admission, 24 and 48 h postoperatively, and at discharge). The effects of “group, time, and group–time interaction” were evaluated. The equality of variances between groups was assessed using Levene’s test, and the homogeneity of covariance matrices was investigated using Box’s M test. A significance level of 0.05 was applied.

## Results

The Chi-square test indicated no significant differences in gender, diabetes, hypertension, and hyperlipidemia between the groups (*P* > 0.05; Table 1). Similarly, an independent samples t-test revealed no significant differences in the mean age, weight, and body mass index (BMI) between the groups (*P* > 0.05). Nonetheless, the average height was significantly higher in the controls in comparison with the intervention group (*P* = 0.023).

**Table 1:** Comparison of demographic characteristics and cardiovascular risk factors in patients according to the study groups

Variable	Group			
	Intervention(n = 31)	Control(n = 31)	Test Statistical Index	*P*-Value
	Frequency/ percentage	Frequency/ percentage		
Gender				
Male	18 (58.1)	22 (71.0)	1.127	0.288
Female	13 (41.9)	9 (29.0)		
Diabetes	16 (51.6)	14 (45.2)	0.258	0.611
Hypertension	25 (80.6)	26 (83.9)	0.111	0.740
Hyperlipidemia	14 (45.2)	12 (38.7)	0.265	0.607
Age (year)	Mean ± SD		− 0.016	0.987
	61.42 ± 7.45	61.45 ± 8.40		
Weight (kg)	76.13 ± 12.35	77.58 ± 8.97	− 0.502	0.617
Height (cm)	165.26 ± 8.36	169.74 ± 6.72	− 2.327	0.023
Body surface area (m^2^)	1.86 ± 0.17	1.91 ± 0.14	− 1.193	0.237

As shown in Table 2, the Fisher’s exact and Chi-square tests displayed no significant differences in the frequency of platelet and blood transfusions in the operating room and ICU between the two groups (*P* > 0.05). Nonetheless, the frequency of fresh frozen plasma (FFP) transfusions in the ICU was significantly lower in the intervention group in comparison with the controls (*P* = 0.010).

**Table 2:** Comparison of the frequency distribution of blood and blood products in patients according to the investigated groups

Variable	Group			
	Intervention (*n* = 31) Frequency (%)	Control (*n* = 31) Frequency (%)	Test Statistical Index	*P*-Value
*Blood transfusion in the operating room (U)*				
0	11 (35.5)	10 (32.2)	0.082	0.960
1	14 (45.2)	15 (48.4)		
> 1	6 (19.4)	6 (19.4)		
*Blood transfusion in the ICU (U)*				
0	25 (80.6)	24 (77.4)	1.112	0.795
1	5 (16.1)	4 (12.9)		
2	1 (3.2)	3 (9.7)		
*Platelet transfusion in the operating room (U)*				
0	14 (45.2)	14 (45.2)	1.202	0.813
1	5 (16.1)	3 (9.7)		
2	10 (32.3)	10 (32.3)		
> 2	2 (6.5)	4 (12.9)		
*Platelet transfusion in the ICU (U)*				
0	26 (83.9)	23 (74.2)	1.036	0.667
1	2 (6.5)	4 (12.9)		
> 1	3 (9.7)	4 (12.9)		
*FFP transfusion in the operating room (Unit)*				
0	16 (51.6)	15 (48.4)	0.416	0.878
1	4 (12.9)	3 (9.7)		
> 1	11 (35.5)	13 (41.9)		
*FFP transfusion in the ICU (U)*				
0	27 (87.1)	18 (58.1)	7.935	0.010
1	0 (0)	5 (16.1)		
> 1	4 (12.9)	8 (25.8)		

ICU, intensive care unit; U, unit; FFP, fresh frozen plasma

As shown in Table 3, the Mann–Whitney U test indicated that the median time to return to baseline heart rate (HR) (in minutes) was significantly shorter in the intervention group in comparison to the controls (*P* = 0.003). Nonetheless, according to Fisher’s exact, the groups displayed no significant difference test in the frequency of inotrope type in the operating room, the need for shock during the procedure, or the requirement for a pacemaker (*P* > 0.05). Additionally, none of the patients in either group needed the implantation of a balloon pump or extracorporeal membrane oxygenation (ECMO).

**Table 3:** Comparison of time to return to baseline heart rate and the need for interventions during and after the end of cardiopulmonary bypass in patients according to the study groups

Variable	Group			
	Intervention (*n* = 31)	Control (*n* = 31)	Test Statistical Index	*P*-Value
Time to return to baseline heart rate (minute)	2 (1.3–5)	4 (5–3)	− 2.003	0.003
Inotrope type in the operating room			0.998	0.999
None	25 (80.6)	24 (72.4)		
Epinephrine	6 (19.4)	6 (19.4)		
Norepinephrine	0 (0)	1 (3.2)		
Need for shock in the operation room	0 (0)	0 (0)	–	0.999
Need for a pacemaker	0 (0)	0 (0)	–	0.999

ANOVA results exhibited a significant group effect (*P* = 0.011), indicating that the mean systolic blood pressure (SBP) had a significant difference between the groups across all time points, with its values being lower in the intervention group. It suggests that vitamin C supplementation reduced SBP, albeit within the normal range.

The analysis also revealed that the time effect was significant (*P* < 0.001), indicating that the average SBP alterations in the groups had a statistically significant difference across different time points. In other words, the mean SBP decreased from anesthesia induction until the end of CPB and then increased.

Statistical analysis also displayed that the group–time interaction was not significant (*P* = 0.201). This means that the slope of the mean SBP changes over the study period had no significant difference between the groups (Table 4).

**Table 4:** Mean, standard deviation, and within-group and between-group effects of systolic blood pressure in the intervention and control groups across all time points

	Group				
Time	Intervention (*n* = 31)	Control (*n* = 31)	Group effect	Time effect	Group–time interaction
Before anesthesia induction	113 ± 12.39	121.1 ± 12.40	F = 6.835 df = 1 *p* = 0.011	F = 16.164 df = 4 *p* < 0.001	F = 1.506 df = 4 *p* = 0.201
After the end of CPB	111.9 ± 12.53	112.8 ± 8.34			
Upon ICU admission	113.7 ± 16.88	121.8 ± 12.32			
12 h after ICU admission	119 ± 13.44	123.2 ± 12.21			
24 h after ICU admission	122.6 ± 12.47	130.2 ± 12.06			

CPB, cardiopulmonary bypass; ICU, intensive care unit

ANOVA results displayed a significant group effect (*P* = 0.002), indicating that the mean diastolic blood pressure (DBP) had a significant difference between the groups across all time points, with its values being lower in the control group. It suggests that vitamin C supplementation reduced DBP, albeit within the normal range.

The analysis also revealed that the time effect was significant (*P* < 0.001), indicating that the mean DBP in the two groups had a significant difference across different time points. In other words, the mean DBP decreased from anesthesia induction until the end of CPB and then increased.

Statistical analysis also displayed that the group–time interaction was not significant (*P* = 0.749). This means that the slope of the mean DBP changes over the study period had no significant difference between the groups (Table 5).

**Table 5:** Mean, standard deviation, and within-group and between-group effects of diastolic blood pressure in the intervention and control groups across all time points

Time	Group				
	Intervention (*n* = 31)	Control (*n* = 31)	Group effect	Time effect	Group–time interaction
Before anesthesia induction	64.5 ± 9.35	71.1 ± 8.77	F = 11.004 df = 1 *p* = 0.002	F = 14.107 df = 4 *p* < 0.001	F = 0.482 df = 4 *p* = 0.749
After the end of CPB	61.3 ± 8.84	64.5 ± 7.00			
Upon ICU admission	64.6 ± 10.87	68.6 ± 6.87			
12 h after ICU admission	65.4 ± 8.88	71.2 ± 9.74			
24 h after ICU admission	70.6 ± 8.34	75.7 ± 10.59			

CPB, cardiopulmonary bypass; ICU, intensive care unit

ANOVA results displayed no significant group effect (*P* = 0.668), indicating that the mean central venous pressure (CVP) had no significant difference between the groups across all time points.

The analysis also revealed that the time effect was significant (*P* < 0.001), indicating that the mean CVP alterations in the two groups had significant differences across different time points. In other words, the mean CVP generally increased from anesthesia induction until 12 h after ICU admission and decreased slightly thereafter.

Statistical analysis also indicated that the group–time interaction was not significant (*P* = 0.510). This means that the slope of the mean CVP changes over the study period had no significant difference between the groups (Table 6).

**Table 6:** Mean, standard deviation, and within-group and between-group effects of central venous pressure in the intervention and control groups across all time points

Time	Group				
	Intervention (*n* = 31)	Control (*n* = 31)	Group effect	Time effect	Group–time interaction
Before anesthesia induction	8.58 ± 1.747	7.84 ± 1.968	F = 0.186 df = 1 *p* = 0.668	F = 40.497 df = 4 *p* < 0.001	F = 0.826 df = 4 *p* = 0.510
After the end of CPB	8.52 ± 2.475	8.19 ± 2.257			
Upon ICU admission	9.42 ± 3.622	9.61 ± 2.591			
12 h after ICU admission	11.58 ± 2.046	12.10 ± 2.427			
24 h after ICU admission	12.00 ± 2.477	11.55 ± 2.501			

CPB, cardiopulmonary bypass; ICU, intensive care unit

ANOVA results revealed no significant group effect (*P* = 0.397), indicating that the mean HR had no significant difference between the intervention and control groups across all time points.

The analysis also revealed that the time effect was significant (*P* < 0.001), indicating that the mean HR changes in the two groups had significant differences across different time points. In other words, the mean HR increased from anesthesia induction thereafter.

Statistical analysis also indicated that the group–time interaction was not significant (*P* = 0.345). This means that the slope of the mean HR alterations over the study period had no significant difference between the groups (Table 7).

**Table 7:** Mean, standard deviation, and within-group and between-group effects of heart rate in the intervention and control groups across all time points

Time	Group				
	Intervention (*n* = 31)	Control (*n* = 31)	Group effect	Time effect	Group–time interaction
Before anesthesia induction	69.23 ± 11.983	68.42 ± 8.921	F = 0.728 df = 1 p = 0.397	F = 37.203 df = 4 p < 0.001	F = 1.125 df = 4 p = 0.345
After the end of CPB	78.74 ± 9.366	74.29 ± 8.513			
Upon ICU admission	80.84 ± 10.696	79.06 ± 8.450			
12 h after ICU admission	81.61 ± 10.579	82.52 ± 10.135			
24 h after ICU admission	83.26 ± 11.069	81.35 ± 7.787			

CPB, cardiopulmonary bypass; ICU, intensive care unit

The t-test displayed that the average duration of the pump and the duration of ventilation in the groups did not have a significant difference (*P* > 0.05), while the average cardiac arrest time (average time to cardiac arrest from the time of cardioplegia injection) in the controls was significantly higher compared to the intervention group (*P* = 0.028). The Mann–Whitney test displayed that the mean duration of hospitalization in the special ward and the normal ward in the groups was not significantly different (*P* > 0.05) (Table 8).

**Table 8:** Comparison of the mean variables during the operation of patients based on the groups under study

Variable	Group			
	Intervention = (31)	Control = (31)	Index value Statistical test	*P*- Value
Pump time (minutes)	74/03 ± 9/44	72/71 ± 7/26	0/624	0/509
Aortic clamp time (minutes)	42/81 ± 6/45	41/22 ± 5/44	1/022	0/311
Ventilation time (hours)	13/35 ± 7/16	11/39 ± 3/77	1/354	0/181
Cardiac arrest time (seconds)	31/52 ± 10/45	37/42 ± 10/16	− 2/255	0/028
Duration of stay in the intensive care unit (days)	2(2–2)	2(2–3)	− 1/346	0/178
Duration of stay in the ward (days)	5(5–5)	5(4–6)	− 0/112	0/911

According to these data, the group effect on CK-MB is not statistically significant; however, the effect of time and the interaction effect of group and time are statistically significant. Thus, the intervention effect of adding vitamin C to the cardioplegia solution, compared to not adding vitamin C, is not significant for CK-MB. Regarding troponin, the data indicate that the group effect and the interaction effect of group and time are not statistically significant, but the effect of time is statistically significant. Thus, the intervention effect of adding vitamin C to the cardioplegia solution, compared to not adding vitamin C, is not significant for troponin (Table 9).

**Table 9:** Effects of CK-MB and troponin in the intervention and control groups based on the time points studied

Time	Group					
	Intervention (*n* = 31)	Control (*n* = 31)	Group effect	Time effect	Group–time interaction	
CK-MB	Before the operation	14/57 ± 5/397	12/02 ± 4/822	F = 0/553	F = 91/811	F = 7/693
	Upon admission to the ICU	30/68 ± 16/220	28/59 ± 9/583	df = 1	df = 2	df = 2
	24 h after ICU admission	32/79 ± 14/321	43/01 ± 19/870	*p* = 0/460	*p* < 0/001	*p* = 0/001
Troponin	Before the operation	12/39 ± 4/887	14/65 ± 5/333	F = 0/188	F = 77/630	F = 0/181
	Upon admission to the ICU	2118 ± 1626/9	2352 ± 1285/1	df = 1	df = 2	df = 2
	24 h after ICU admission	2001 ± 1692/7	2074 ± 1712/1	*p* = 0/666	*p* < 0/001	*p* = 0/835

## Discussion

Coronary artery disease (CAD) is the leading cause of death in the current century, and cardiac surgery plays a vital role in human health [19, 20]. The results regarding hemodynamic parameters before anesthesia induction, after cardioplegic injection, during rewarming, after the end of CPB, upon ICU admission, and 12 and 24 h postoperatively in CABG cases receiving CPB in the control and intervention groups was investigated. According to the obtained results, the time effect was statistically significant for all hemodynamic parameters. For CVP and HR, neither the group effect nor the group–time interaction was statistically significant. Therefore, the interventional effect of adding vitamin C to the cardioplegic solution, compared to not adding vitamin C, on these variables was not significant, and the corresponding hypothesis was rejected. The group effect on SBP and DBP was statistically significant. Thus, the interventional effect of adding vitamin C to the cardioplegic solution, compared to not adding vitamin C, on these variables was significant, supporting the hypothesis in this regard. Most of the reviewed articles did not provide information regarding hemodynamic parameters. However, a systematic review by Hill et al.[8] referred to another study, where the administration of 3 g of vitamin C 12 to 18 h prior to operation and the same amount during surgery displayed no significant difference in blood gases between the two groups. In Emadi et al.’s [18] study at Shiraz University of Medical Sciences and Ebade et al.’s [21] study in Cairo, entitled “Comparison of Ascorbic Acid with Magnesium for the Prevention of Atrial Fibrillation after Coronary Artery Bypass Grafting,” the hemodynamic parameters assessed in the control and intervention groups had no significant difference. The results of these studies can be considered both consistent and inconsistent with our study in certain aspects.

The results of our study demonstrated no significant difference concerning the average duration of ventilation between the groups. Therefore, it can be said that the intervention of adding vitamin C to the cardioplegic solution, compared to not adding vitamin C, had no impact on the participants’ mean duration of intubation, and the corresponding hypothesis is also rejected. In Emadi et al.’s [18] study at Shiraz University of Medical Sciences, vitamin C did not make a difference in the mean duration of intubation. Similarly, in Ebade et al.’s [21] study in Cairo, entitled “Comparison of Ascorbic Acid with Magnesium for the Prevention of Atrial Fibrillation after Coronary Artery Bypass Grafting,” there were no significant differences regarding the average duration of ventilation between the control and intervention groups. In another study conducted by Hill et al. [8] in Aachen, Germany, no significant difference was found in the average length of ventilation in patients who had preoperative vitamin C levels below the optimal range or in those whose vitamin C levels decreased by more than 50% postoperatively. Their results align with the findings of the current research. In a study conducted at Tabriz University of Medical Sciences, Safaei et al. [22] compared the effects of grape seed oil and ascorbic acid on OS induced by CPB in patients undergoing CABG. They found a significant difference regarding the average length of ventilation between the two intervention groups and the control group, with the intervention groups demonstrating a shorter duration of mechanical ventilation compared to the controls. Their results contradict our study, and the discrepancy in results may be due to differences in the methodology. In Hill et al.’s [8] systematic review, no significant difference was detected between the two groups in average duration of ventilation. These results align with our study results. However, Hill et al.’s [8] systematic review refers to another study conducted by Sadeghpour [23], which declared that vitamin C enhanced the length of ventilation. The results of this study are not consistent with ours. The discrepancy in results may be related to variations in the methodology, as in this research, vitamin C was administered at 2 g prior to surgery and 1 g daily for up to 4 days post-surgery. A total of 290 patients, patients undergoing both CABG and valve surgery, participated in this research.

Additionally, there were no significant differences in the length of ICU and general ward stay between the groups. So, the hypothesis in this regard is not supported. A study conducted in 2017 in Maribor, Slovenia, failed to find a significant difference in the mean duration of ICU and hospital stay between the controls and the vitamin C-receiving group. Hill et al. [8] in Aachen, Germany, indicated no significant difference in the average duration of ICU and hospital stay between patients who had suboptimal vitamin C concentrations before surgery or those who experienced a greater than 50% reduction in vitamin C concentrations post-surgery. In a study conducted at Tabriz University of Medical Sciences, Safaei et al. [21] compared the impacts of grape seed oil and ascorbic acid on OS induced by CPB in patients undergoing CABG and found no significant difference in the average length of ICU stay between the intervention and control groups. The findings of this study are consistent with ours. In Emadi et al.’s [18] study at Shiraz University of Medical Sciences, vitamin C did not significantly influence the average length of stay in hospital; however, it was found to reduce the average length of ICU stay. Hill et al.’s [8] systematic review refers to another study, which found that vitamin C shortened the length of stay in hospital but did not affect the ICU stay duration. Furthermore, this systematic review refers to other studies that found that vitamin C reduced the length of ICU and hospital stay. However, a study did not find a significant difference between the two groups in the mean length of ICU and hospital stay. In Ebade et al.’s [21] study in Cairo, entitled “Comparison of Ascorbic Acid with Magnesium for the Prevention of Atrial Fibrillation after Coronary Artery Bypass Grafting,” patients in the control group showed significantly longer lengths of ICU and hospital stay in comparison with both the ascorbic acid and magnesium groups. This difference between the two intervention groups was not significant.

Regarding troponin, according to the data, the group effect and the interaction effect of group and time are not statistically significant, but the effect of time is statistically significant. Thus, the intervention effect of adding vitamin C to the cardioplegia solution, compared to not adding vitamin C, is not significant for troponin. Consequently, the related hypothesis is also rejected. In the study by Emadi and colleagues at Shiraz University of Medical Sciences, the researchers also found similar results [18]. The results of this study align with those obtained in our study. In the systematic review by Hill and colleagues in 2018 [8], vitamin C was shown to reduce CK-MB, but its effect on troponin was not addressed. The results of this study are inconsistent with those obtained in our study. The difference in results may be due to variations in the execution method, as in that study, 125 mg of vitamin C per kilogram of body weight was injected before and after cardiopulmonary bypass.

## Conclusion

The findings of the current research demonstrate that adding vitamin C to the cardioplegic solution can provide benefits, such as reduced cardiac arrest time (average time to cardiac arrest from the time of cardioplegia injection) and return to baseline HR, as well as decreased plasma requirements in the ICU. Additionally, adding vitamin C to the cardioplegic solution can maintain higher sodium levels and reduce blood pressure. According to the results, the implemented intervention has no significant effect on other investigated parameters. Due to the low cost and availability of vitamin C, it seems that adding vitamin C to the cardioplegic solution, with its minimal positive effects and no negative side effects, can be performed.

## Data Availability

The data are available by corresponding authors as requested.

## Abbreviations

CPB: Cardiopulmonary bypass
CABG: Coronary artery bypass graft
CVP: Central venous pressure
SBP: Systolic blood pressure
DBP: Diastolic blood pressure
